# The genome sequence of the Judas Tree Seed Beetle, *Bruchidius siliquastri *Delobel, 2007

**DOI:** 10.12688/wellcomeopenres.21109.1

**Published:** 2024-03-11

**Authors:** Maxwell V. L. Barclay, Michael Geiser, Keita Matsumoto, Emily Pash, Natural History Museum Genome Acquisition Lab, Darwin Tree of Life Barcoding collective, Wellcome Sanger Institute Tree of Life Management, Samples and Laboratory team, Wellcome Sanger Institute Scientific Operations: Sequencing Operations, Wellcome Sanger Institute Tree of Life Core Informatics team, Tree of Life Core Informatics collective, Darwin Tree of Life Consortium

**Affiliations:** 1 Natural History Museum, London, England, UK

**Keywords:** Bruchidius siliquastri, the Judas tree Seed Beetle, genome sequence, chromosomal, Coleoptera

## Abstract

We present a genome assembly from an individual male *Bruchidius siliquastri* (the Judas tree Seed Beetle; Arthropoda; Insecta; Coleoptera; Chrysomelidae). The genome sequence is 375.6 megabases in span. Most of the assembly is scaffolded into 11 chromosomal pseudomolecules, including the X and Y sex chromosomes. The mitochondrial genome has also been assembled and is 15.81 kilobases in length. Gene annotation of this assembly on Ensembl identified 17,940 protein coding genes.

## Species taxonomy

Eukaryota; Opisthokonta; Metazoa; Eumetazoa; Bilateria; Protostomia; Ecdysozoa; Panarthropoda; Arthropoda; Mandibulata; Pancrustacea; Hexapoda; Insecta; Dicondylia; Pterygota; Neoptera; Endopterygota; Coleoptera; Polyphaga; Cucujiformia; Chrysomeloidea; Chrysomelidae; Bruchinae; Bruchini; *Bruchidius*; *Bruchidius siliquastri* Delobel, 2007 (NCBI:txid1649775).

## Background

*Bruchidius siliquastri* was described as new to science based on specimens collected in southern France by Delobel in Kergoat *et al.* (2007), who also cite specimens from Hungary and China (not included in the type series). They assume that the species was imported to Europe from China. It has since been reported from Slovakia ( Kollár *et al.*, 2009), Belgium ( Hanssens, 2009), the Czech Republic ( Šefrova *et al.*, 2010), Bulgaria ( Stojanova *et al.*, 2011), Serbia ( Gavrilović & Savić, 2013), Germany ( Rheinheimer & Hassler, 2013), Turkey ( Hizal & Parlak, 2013), Ukraine ( Martynov & Nikulina, 2015), Italy ( Yus Ramos & Bocci, 2017), Romania ( Pintilioaie *et al.*, 2018), Korea ( Jeong *et al.*, 2022), as well as from Spain, where its life history and immature stages have been explored ( Yus Ramos *et al.*, 2009a; Yus Ramos *et al.*, 2009b; Yus Ramos *et al.*, 2009c). It was first reported in Britain from South Kensington in 2014 ( Barclay, 2014) and has since become widespread in the London area and in other cities and towns in southern Britain. Although Britain is the northern limit of its currently known distribution, the beetle is actively expanding its range, and trees where it was apparently absent 5 to 10 years ago now support populations.

*B. siliquastri* develops in the seed pods of leguminous trees of the genus *Cercis* (Fabaceae), particularly the Judas tree *Cercis siliquastrum* Linnaeus. *Cercis* occurs throughout the northern hemisphere, and while the Judas tree is native to southern Europe and western Asia, it is a popular planted ornamental in cities and parks elsewhere because of its bright flowers and attractive foliage. It is common in the Levant, and may owe its name to a legend that Judas Iscariot hanged himself from one ( Barclay, 2014), but the majority of London specimens are much too small for this. An alternative theory is that the name is a corruption of ‘Judea’, the apparent origin of European specimens. The tree features in Middle Eastern art and poetry, and the edible flowers and young leaves are sometimes used as a culinary garnish, but there is no indication that the beetle was present in Europe or Western Asia until the 2000s.

Adults of *B. siliquastri* are most easily collected by beating flowers, pods and foliage of *Cercis* from May to August, but they can also be reared from pods collected in spring. Stojanova *et al.* (2011) observed a large parasitoid fauna associated with *Bruchidius siliquastri* in Bulgaria, but no species of Hymenoptera have been reared from the London population of this beetle so far. The only other herbivorous insect recorded feeding on the trees was the jumping plant louse *Cacopsylla pulchella* (Löw) (Hemiptera: Psyllidae).

*Bruchidius siliquastri* can immediately be recognised from nearly all other species of Bruchinae occurring in Northern Europe by its bright red abdomen. This is conspicuous when the beetle is on its back, but is also visible dorsally, since the red pygidium extends well beyond the apices of the elytra. Apart from this striking feature it resembles a slightly small example of the common *Bruchidius villosus* (Fabricius), being black with whitish pubescence on the dorsal surface. The only other European Bruchinae with a reddish abdomen is the cosmopolitan *Acanthoscelides obtectus* (Say), an occasional kitchen and warehouse pest associated with dried beans. *Acanthoscelides* can resemble a large *Bruchidius* when stored in ethanol, but immediately differs by the dense brownish vestiture of elytra and pronotum when alive or dry. It is also immediately recognisable by the sharp teeth on the hind femora, along with its broader shape ( Duff, 2016).

The specimens used for the Darwin Tree of Life project were collected on 23 July 2021 by a small Natural History Museum team, conducting a survey of Wetherby Gardens at the request of Natural History Museum patrons Holly Smith and Neil Osborn. The beetles were beaten in large numbers from a small Judas Tree growing from the pavement near the entrance to Wetherby Garden Square, less than a mile from the original South Kensington locality ( Barclay, 2014).

## Genome sequence report

**Figure 1.**
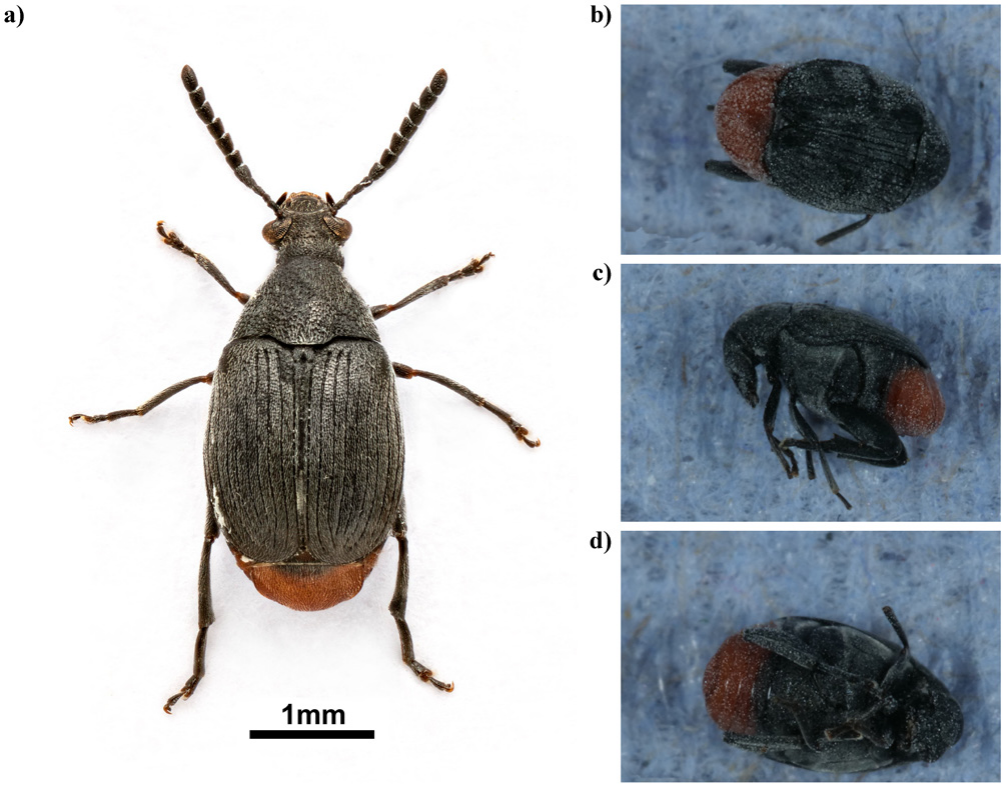
Photographs of *Bruchidius siliquastri*: **a**) A specimen collected in Kensington in 2014: photograph by Harry Taylor (Natural History Museum); **b**)– **d**) The specimen used for genome sequencing (icBruSili1): **b**) dorsal view, **c**) lateral view and d) ventral view.

The genome was sequenced from one male *Bruchidius siliquastri* ( Figure 1) collected from Wetherby Gardens, Kensington, London, UK (51.49, –0.18). A total of 57-fold coverage in Pacific Biosciences single-molecule HiFi long reads was generated. Primary assembly contigs were scaffolded with chromosome conformation Hi-C data. Manual assembly curation corrected 26 missing joins or mis-joins and removed 6 haplotypic duplications, reducing the assembly length by 1.11% and the scaffold number by 44.83%, and increasing the scaffold N50 by 10.13%.

**Figure 2.**
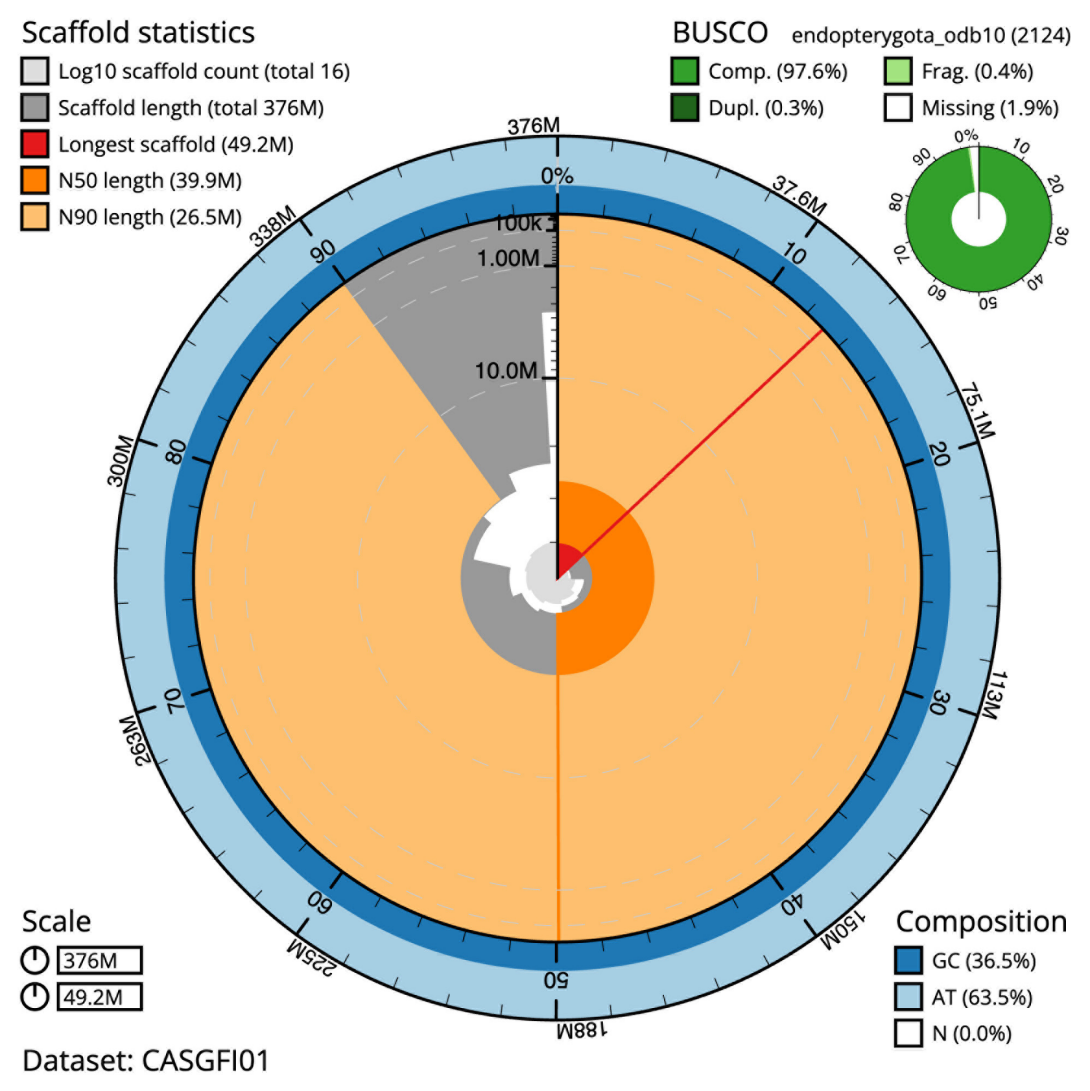
Genome assembly of *Bruchidius siliquastri*, icBruSili1.1: metrics. The BlobToolKit Snailplot shows N50 metrics and BUSCO gene completeness. The main plot is divided into 1,000 size-ordered bins around the circumference with each bin representing 0.1% of the 375,583,928 bp assembly. The distribution of scaffold lengths is shown in dark grey with the plot radius scaled to the longest scaffold present in the assembly (49,182,353 bp, shown in red). Orange and pale-orange arcs show the N50 and N90 scaffold lengths (39,857,073 and 26,473,722 bp), respectively. The pale grey spiral shows the cumulative scaffold count on a log scale with white scale lines showing successive orders of magnitude. The blue and pale-blue area around the outside of the plot shows the distribution of GC, AT and N percentages in the same bins as the inner plot. A summary of complete, fragmented, duplicated and missing BUSCO genes in the endopterygota_odb10 set is shown in the top right. An interactive version of this figure is available at https://blobtoolkit.genomehubs.org/view/CASGFI01/dataset/CASGFI01/snail.

**Figure 3.**
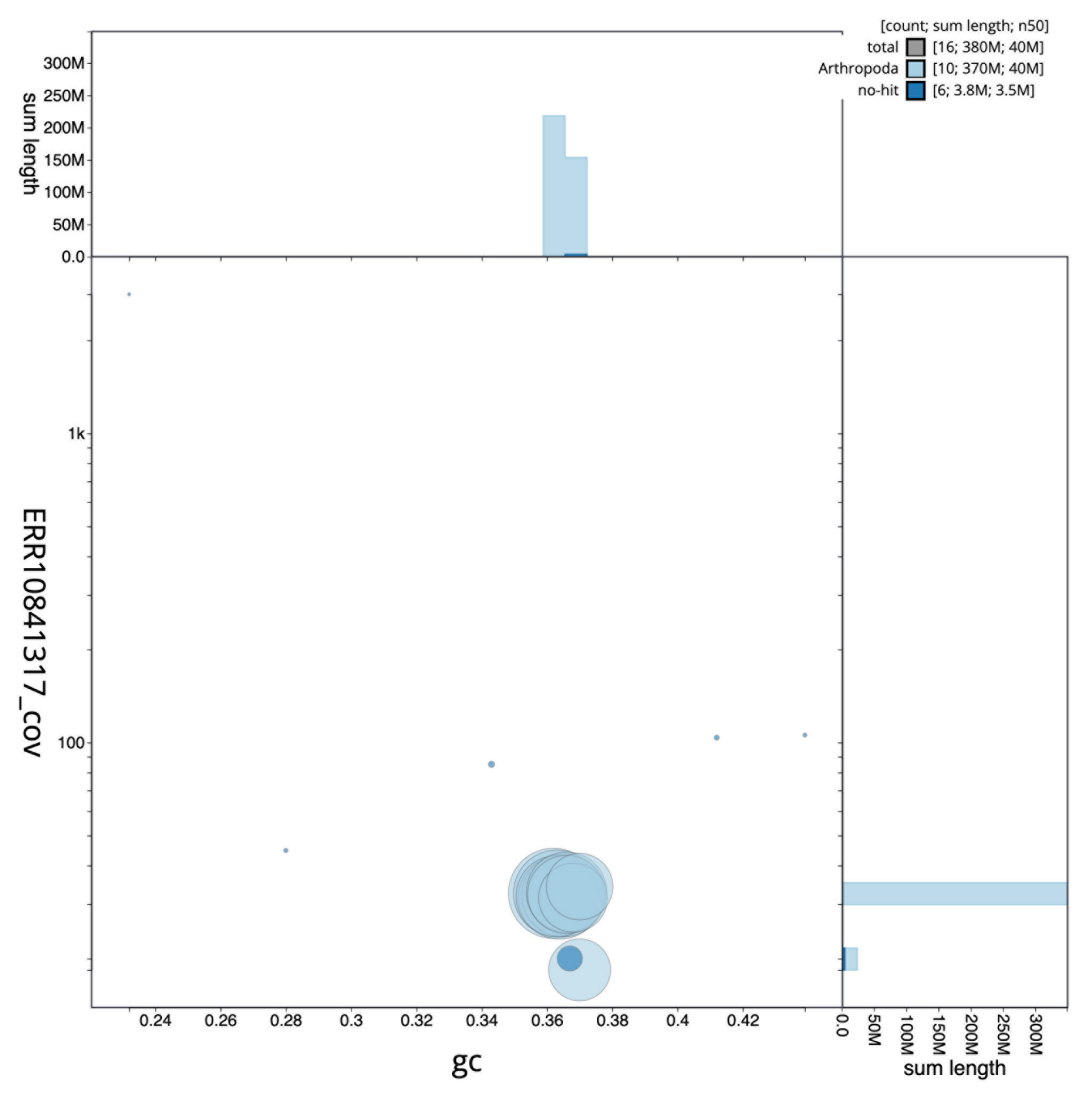
Genome assembly of *Bruchidius siliquastri*, icBruSili1.1: BlobToolKit GC-coverage plot. Scaffolds are coloured by phylum. Circles are sized in proportion to scaffold length. Histograms show the distribution of scaffold length sum along each axis. An interactive version of this figure is available at https://blobtoolkit.genomehubs.org/view/CASGFI01/dataset/CASGFI01/blob.

**Figure 4.**
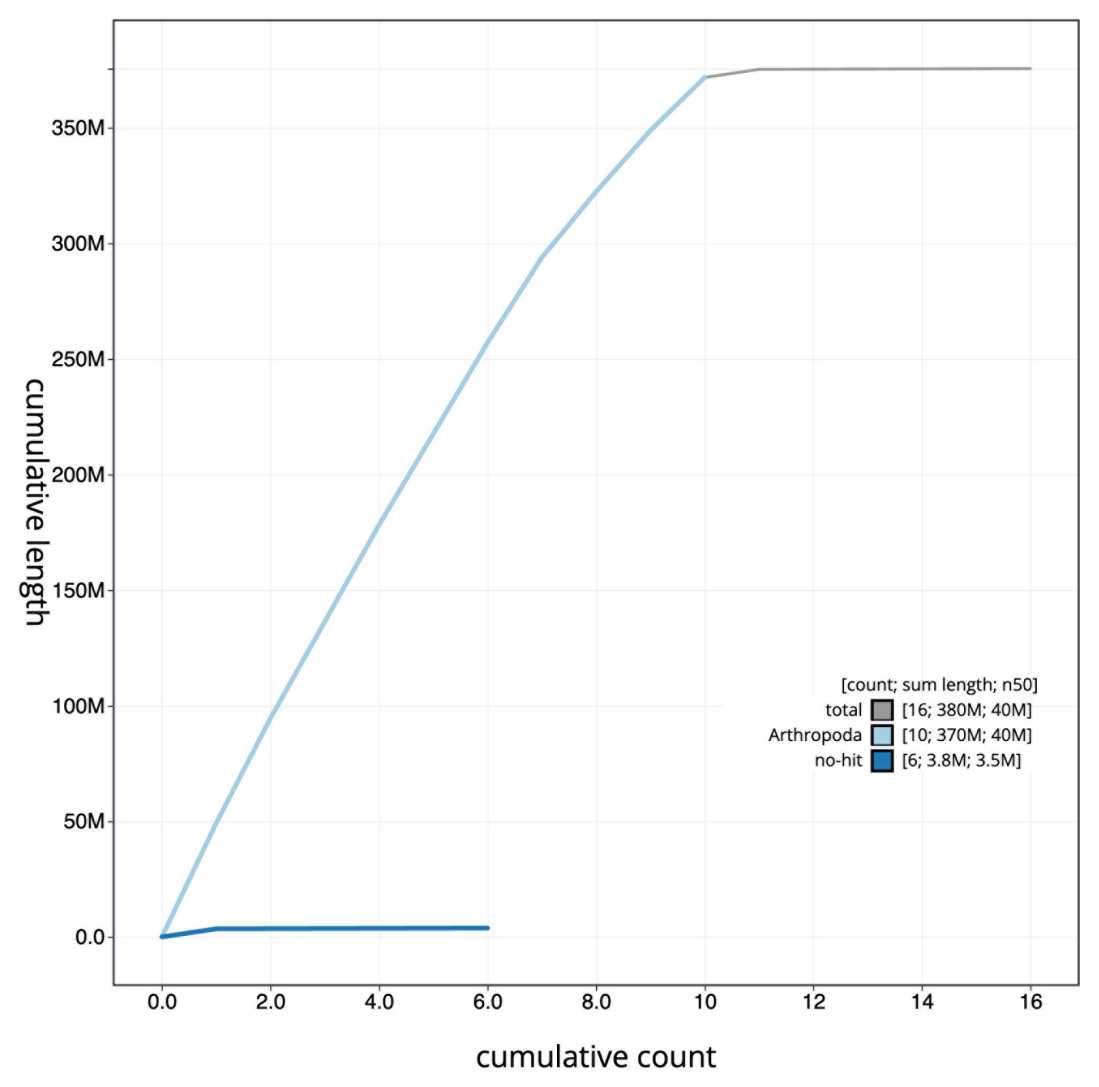
Genome assembly of *Bruchidius siliquastri*, icBruSili1.1: BlobToolKit cumulative sequence plot. The grey line shows cumulative length for all scaffolds. Coloured lines show cumulative lengths of scaffolds assigned to each phylum using the buscogenes taxrule. An interactive version of this figure is available at https://blobtoolkit.genomehubs.org/view/CASGFI01/dataset/CASGFI01/cumulative.

**Figure 5.**
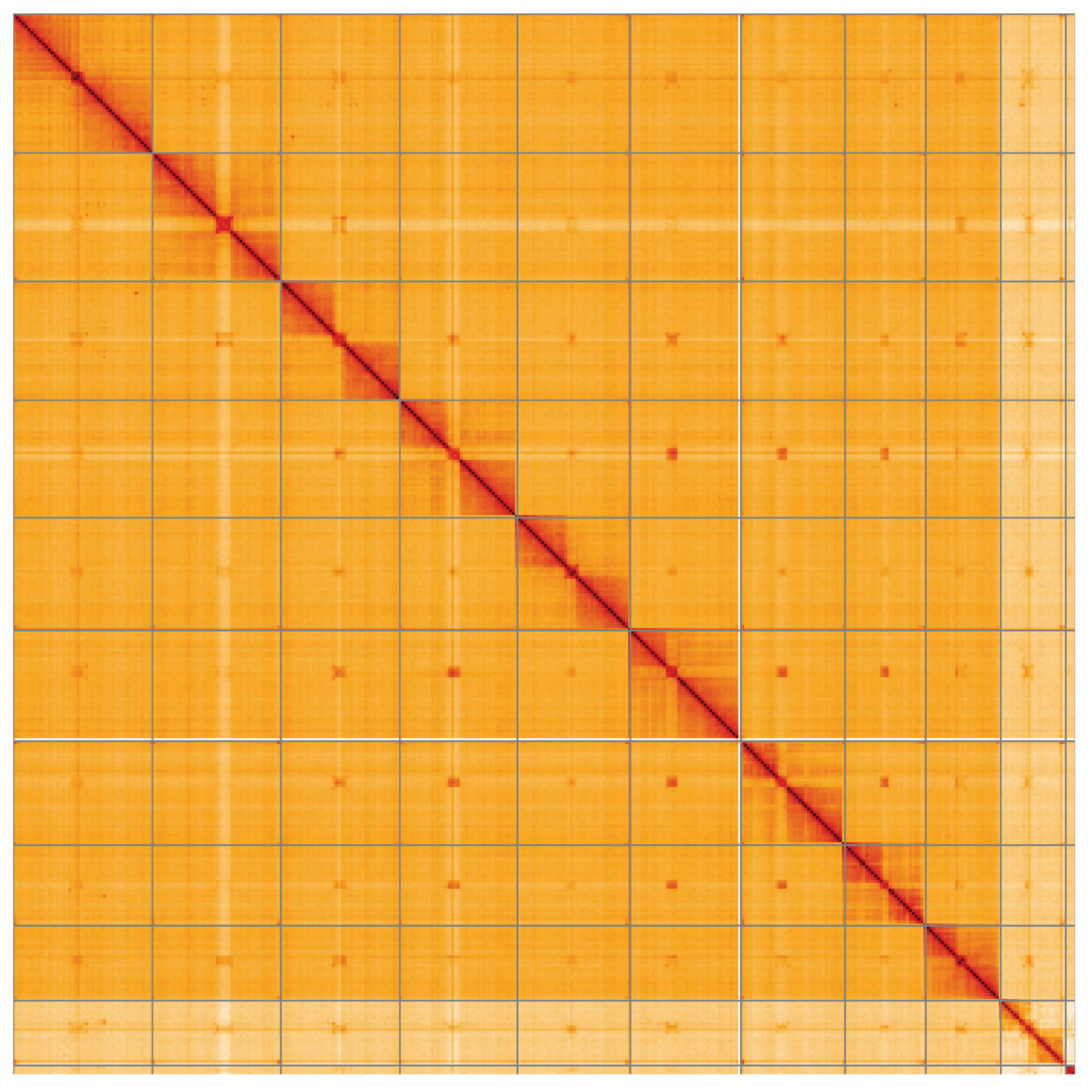
Genome assembly of *Bruchidius siliquastri*, icBruSili1.1: Hi-C contact map of the icBruSili1.1 assembly, visualised using HiGlass. Chromosomes are shown in order of size from left to right and top to bottom. An interactive version of this figure may be viewed at https://genome-note-higlass.tol.sanger.ac.uk/l/?d=ff3I2oGrSVaZWymS1fyS7w.

The final assembly has a total length of 375.6 Mb in 15 sequence scaffolds with a scaffold N50 of 39.9 Mb ( Table 1). The snailplot in Figure 2 provides a summary of the assembly statistics, while the distribution of assembly scaffolds on GC proportion and coverage is shown in Figure 3. The cumulative assembly plot in Figure 4 shows curves for subsets of scaffolds assigned to different phyla. Most (99.95%) of the assembly sequence was assigned to 11 chromosomal-level scaffolds, representing 9 autosomes and the X and Y sex chromosomes. Chromosome-scale scaffolds confirmed by the Hi-C data are named in order of size ( Figure 5; Table 2). While not fully phased, the assembly deposited is of one haplotype. Contigs corresponding to the second haplotype have also been deposited. The mitochondrial genome was also assembled and can be found as a contig within the multifasta file of the genome submission.

**Table 1.** Genome data for *Bruchidius siliquastri*, icBruSili1.1.

Project accession data		
Assembly identifier	icBruSili1.1	
Species	*Bruchidius siliquastri*	
Specimen	icBruSili1	
NCBI taxonomy ID	1649775	
BioProject	PRJEB59372	
BioSample ID	SAMEA111458828	
Isolate information	icBruSili1, male: abdomen (DNA sequencing); head and thorax (Hi-C sequencing)	
Assembly metrics *		*Benchmark*
Consensus quality (QV)	60.3	*≥ 50*
*k*-mer completeness	100.0%	*≥ 95%*
BUSCO **	C:97.6%[S:97.4%,D:0.3%], F:0.4%,M:1.9%,n:2,124	*C ≥ 95%*
Percentage of assembly mapped to chromosomes	99.95%	*≥ 95%*
Sex chromosomes	XY	*localised homologous pairs*
Organelles	Mitochondrial genome: 15.81 kb	*complete single alleles*
Raw data accessions		
PacificBiosciences SEQUEL II	ERR10841317	
Hi-C Illumina	ERR10851510	
Genome assembly		
Assembly accession	GCA_949316355.1	
*Accession of alternate haplotype*	GCA_949316485.1	
Span (Mb)	375.6	
Number of contigs	87	
Contig N50 length (Mb)	7.0	
Number of scaffolds	15	
Scaffold N50 length (Mb)	39.9	
Longest scaffold (Mb)	49.18	
Genome annotation		
Number of protein-coding genes	17,940	
Number of gene transcripts	18,096	

* Assembly metric benchmarks are adapted from column VGP-2020 of “Table 1: Proposed standards and metrics for defining genome assembly quality” from ( Rhie *et al.*, 2021). ** BUSCO scores based on the endopterygota_odb10 BUSCO set using version 5.3.2. C = complete [S = single copy, D = duplicated], F = fragmented, M = missing, n = number of orthologues in comparison. A full set of BUSCO scores is available at https://blobtoolkit.genomehubs.org/view/CASGFI01/dataset/CASGFI01/busco.

**Table 2.** Chromosomal pseudomolecules in the genome assembly of *Bruchidius siliquastri*, icBruSili1.

INSDC accession	Chromosome	Length (Mb)	GC%
OX438529.1	1	49.18	36.0
OX438530.1	2	45.38	36.5
OX438531.1	3	42.04	36.5
OX438532.1	4	41.56	36.5
OX438533.1	5	39.86	36.5
OX438534.1	6	39.04	36.5
OX438535.1	7	36.82	36.5
OX438536.1	8	28.49	37.0
OX438537.1	9	26.47	37.0
OX438538.1	X	22.91	37.0
OX438539.1	Y	3.5	36.5
OX438540.1	MT	0.02	23.0

The estimated Quality Value (QV) of the final assembly is 60.3 with *k*-mer completeness of 100.0%, and the assembly has a BUSCO v5.3.2 completeness of 97.6% (single = 97.4%, duplicated = 0.3%), using the endopterygota_odb10 reference set ( *n* = 2,124).

Metadata for specimens, barcode results, spectra estimates, sequencing runs, contaminants and pre-curation assembly statistics are given at https://links.tol.sanger.ac.uk/species/1649775.

## Genome annotation report

The *Bruchidius siliquastri* genome assembly (GCA_949316355.1) was annotated using the Ensembl rapid annotation pipeline at the European Bioinformatics Institute (EBI). The resulting annotation includes 18,096 transcribed mRNAs from 17,940 protein-coding genes ( Table 1; https://rapid.ensembl.org/Bruchidius_siliquastri_GCA_949316355.1/Info/Index)..

## Methods

### Sample acquisition and nucleic acid extraction

A male *Bruchidius siliquastri* (specimen ID NHMUK014440509, ToLID icBruSili1) was handpicked from Wetherby Gardens, Kensington, London, UK (latitude 51.49, longitude –0.18) on 2021-07-23. The specimen was collected by Maxwell Barclay, Michael Geiser, Keita Matsumoto and Emily Pash (Natural History Museum) and identified by Maxwell Barclay, and then preserved by dry freezing at –80 °C.

The workflow for high molecular weight (HMW) DNA extraction at the WSI includes a sequence of core procedures: sample preparation; sample homogenisation, DNA extraction, fragmentation, and clean-up. In sample preparation, the icBruSili1 sample was weighed and dissected on dry ice ( Jay *et al.*, 2023). Tissue from the abdomen was homogenised using a PowerMasher II tissue disruptor ( Denton *et al.*, 2023a). HMW DNA was extracted in the WSI Scientific Operations core using the Automated MagAttract v2 protocol ( Oatley *et al.*, 2023). HMW DNA was sheared into an average fragment size of 12–20 kb in a Megaruptor 3 system with speed setting 31 ( Bates *et al.*, 2023). Sheared DNA was purified by solid-phase reversible immobilisation ( Strickland *et al.*, 2023): in brief, the method employs a 1.8X ratio of AMPure PB beads to sample to eliminate shorter fragments and concentrate the DNA. The concentration of the sheared and purified DNA was assessed using a Nanodrop spectrophotometer and Qubit Fluorometer and Qubit dsDNA High Sensitivity Assay kit. Fragment size distribution was evaluated by running the sample on the FemtoPulse system.

Protocols developed by the Wellcome Sanger Institute (WSI) Tree of Life core laboratory have been deposited on protocols.io ( Denton *et al.*, 2023b).

### Sequencing

Pacific Biosciences HiFi circular consensus DNA sequencing libraries were constructed according to the manufacturers’ instructions. DNA sequencing was performed by the Scientific Operations core at the WSI on a Pacific Biosciences SEQUEL II instrument. Hi-C data were also generated from head and thorax tissue of icBruSili1 using the Arima2 kit and sequenced on the Illumina NovaSeq 6000 instrument.

### Genome assembly, curation and evaluation

Assembly was carried out with Hifiasm ( Cheng *et al.*, 2021) and haplotypic duplication was identified and removed with purge_dups ( Guan *et al.*, 2020). The assembly was then scaffolded with Hi-C data ( Rao *et al.*, 2014) using YaHS ( Zhou *et al.*, 2023). The assembly was checked for contamination and corrected as described previously ( Howe *et al.*, 2021). Manual curation was performed using HiGlass ( Kerpedjiev *et al.*, 2018) and Pretext ( Harry, 2022). The mitochondrial genome was assembled using MitoHiFi ( Uliano-Silva *et al.*, 2023), which runs MitoFinder ( Allio *et al.*, 2020) or MITOS ( Bernt *et al.*, 2013) and uses these annotations to select the final mitochondrial contig and to ensure the general quality of the sequence.

A Hi-C map for the final assembly was produced using bwa-mem2 ( Vasimuddin *et al.*, 2019) in the Cooler file format ( Abdennur & Mirny, 2020). To assess the assembly metrics, the *k*-mer completeness and QV consensus quality values were calculated in Merqury ( Rhie *et al.*, 2020). This work was done using Nextflow ( Di Tommaso *et al.*, 2017) DSL2 pipelines “sanger-tol/readmapping” ( Surana *et al.*, 2023a) and “sanger-tol/genomenote” ( Surana *et al.*, 2023b). The genome was analysed within the BlobToolKit environment ( Challis *et al.*, 2020) and BUSCO scores ( Manni *et al.*, 2021; Simão *et al.*, 2015) were calculated.

Table 3 contains a list of relevant software tool versions and sources.

**Table 3.** Software tools: versions and sources.

Software tool	Version	Source
BlobToolKit	4.1.7	https://github.com/blobtoolkit/blobtoolkit
BUSCO	5.3.2	https://gitlab.com/ezlab/busco
Hifiasm	0.16.1-r375	https://github.com/chhylp123/hifiasm
HiGlass	1.11.6	https://github.com/higlass/higlass
Merqury	MerquryFK	https://github.com/thegenemyers/MERQURY. FK
MitoHiFi	2	https://github.com/marcelauliano/MitoHiFi
PretextView	0.2	https://github.com/wtsi-hpag/PretextView
purge_dups	1.2.3	https://github.com/dfguan/purge_dups
sanger-tol/ genomenote	v1.0	https://github.com/sanger-tol/genomenote
sanger-tol/ readmapping	1.1.0	https://github.com/sanger-tol/readmapping/ tree/1.1.0
YaHS	1.2a	https://github.com/c-zhou/yahs

### Genome annotation

The BRAKER2 pipeline ( Brůna *et al.*, 2021) was used in the default protein mode to generate annotation for the *Bruchidius siliquastri* assembly (GCA_949316355.1) in Ensembl Rapid Release at the EBI.

### Wellcome Sanger Institute – Legal and Governance

The materials that have contributed to this genome note have been supplied by a Darwin Tree of Life Partner. The submission of materials by a Darwin Tree of Life Partner is subject to the **‘Darwin Tree of Life Project Sampling Code of Practice’**, which can be found in full on the Darwin Tree of Life website here. By agreeing with and signing up to the Sampling Code of Practice, the Darwin Tree of Life Partner agrees they will meet the legal and ethical requirements and standards set out within this document in respect of all samples acquired for, and supplied to, the Darwin Tree of Life Project.

Further, the Wellcome Sanger Institute employs a process whereby due diligence is carried out proportionate to the nature of the materials themselves, and the circumstances under which they have been/are to be collected and provided for use. The purpose of this is to address and mitigate any potential legal and/or ethical implications of receipt and use of the materials as part of the research project, and to ensure that in doing so we align with best practice wherever possible. The overarching areas of consideration are:

•       Ethical review of provenance and sourcing of the material

•       Legality of collection, transfer and use (national and international)

Each transfer of samples is further undertaken according to a Research Collaboration Agreement or Material Transfer Agreement entered into by the Darwin Tree of Life Partner, Genome Research Limited (operating as the Wellcome Sanger Institute), and in some circumstances other Darwin Tree of Life collaborators.

## Data Availability

European Nucleotide Archive: *Bruchidius siliquastri*. Accession number PRJEB59372; https://identifiers.org/ena.embl/PRJEB59372 ( Wellcome Sanger Institute, 2023). The genome sequence is released openly for reuse. The *Bruchidius siliquastri* genome sequencing initiative is part of the Darwin Tree of Life (DToL) project. All raw sequence data and the assembly have been deposited in INSDC databases. Raw data and assembly accession identifiers are reported in Table 1.

## Author information

Members of the Natural History Museum Genome Acquisition Lab are listed here: https://doi.org/10.5281/zenodo.7139035.

Members of the Darwin Tree of Life Barcoding collective are listed here: https://doi.org/10.5281/zenodo.4893703.

Members of the Wellcome Sanger Institute Tree of Life Management, Samples and Laboratory team are listed here: https://doi.org/10.5281/zenodo.10066175.

Members of Wellcome Sanger Institute Scientific Operations: Sequencing Operations are listed here: https://doi.org/10.5281/zenodo.10043364.

Members of the Wellcome Sanger Institute Tree of Life Core Informatics team are listed here: https://doi.org/10.5281/zenodo.10066637.

Members of the Tree of Life Core Informatics collective are listed here: https://doi.org/10.5281/zenodo.5013541.

Members of the Darwin Tree of Life Consortium are listed here: https://doi.org/10.5281/zenodo.4783558.
