# Supplementary material for: The genome sequence of the Judas Tree Seed Beetle, Bruchidius siliquastri Delobel, 2007
Source: Wellcome Open Res. 2024 Mar 11;9:142. [Version 1] doi: 10.12688/wellcomeopenres.21109.1 (PMC11316170; doi:10.12688/wellcomeopenres.21109.1)
Supplement: Peer review history

# Reviewer report: Reviewer response for version 1

**DOI:** 10.21956/wellcomeopenres.23349.r90480

**Published:** 2024-08-09

**Authors:** Doga Cedden

**Affiliations:** 1 Department of Evolutionary Developmental Genetics, Johann-Friedrich-Blumenbach Institute, Göttingen, Germany

Barclay et al. report a high-quality genome assembly from *Bruchidius siliquastri. *The background information about the beetle is adequately covered in the data note. The method is sufficiently described and appropriate, and the authors also present a sufficient quality assessment of the assembly. The reported assembly is available in the provided depository.

**I have a single suggestion:**

 The *Bruchidius siliquastri *is a bean weevil (Bruchinae). It would be easier for readers if bean weevil (in its English form) is mentioned at least once in the background.

Are sufficient details of methods and materials provided to allow replication by others?

Yes

Is the rationale for creating the dataset(s) clearly described?

Yes

Are the datasets clearly presented in a useable and accessible format?

Yes

Are the protocols appropriate and is the work technically sound?

Yes

Reviewer Expertise:

I work on coleopteran pests using RNA interference and RNA-seq methods.

I confirm that I have read this submission and believe that I have an appropriate level of expertise to confirm that it is of an acceptable scientific standard.

# Reviewer report: Reviewer response for version 1

**DOI:** 10.21956/wellcomeopenres.23349.r82789

**Published:** 2024-05-21

**Authors:** Liang Lü

**Affiliations:** 1 Hebei Normal University, Shijiazhuang, China

I have read the main text of the manuscript, which reports a recently described seed-beetle, Bruchidius siliquastri Delobel, 2007. It is a satisfying paper that publishes an NGS sequence with detailed description of the background of the sample information in taxonomy, biology, collection, etc. The authors kindly presented in this manuscript clear images that make their identification trustable. The workflows of sample preparation, DNA extraction, sequencing, assembly, and annotation are fine and acceptable, with a few points of imperfection:

 1. I suggest the authors integrate the duplicated descriptions (in the Genome sequence report and the Methods) of the sample information.

 2. The authors refer the sequenced part of the sample is the "tissue from the abdomen", which tissue(s) were isolated for sequencing, muscle or gut or all of soft tissues? If the authors used the later two, how did they do with the DNA of dwelling microbes?

 3. The use of BRAKER2, a pipeline includes many tools, seemed not to be as fully described as the programs used in previous procedures.

Are sufficient details of methods and materials provided to allow replication by others?

Yes

Is the rationale for creating the dataset(s) clearly described?

Yes

Are the datasets clearly presented in a useable and accessible format?

Yes

Are the protocols appropriate and is the work technically sound?

Yes

Reviewer Expertise:

rove beetle taxonomy and molecular evolution

I confirm that I have read this submission and believe that I have an appropriate level of expertise to confirm that it is of an acceptable scientific standard.
